# Complete mitogenome and intra-family comparative mitogenomics showed distinct position of Pama Croaker *Otolithoides pama*

**DOI:** 10.1038/s41598-024-64791-1

**Published:** 2024-06-15

**Authors:** Most Ayesha Siddika, Khandaker Asif Ahmed, Mohammad Shamimul Alam, Jannatul Bushra, Rowshan Ara Begum

**Affiliations:** 1 Genetics and Molecular Biology Laboratory, Department of Zoology, University of Dhaka, Dhaka, 1000 Bangladesh; 2 CSIRO Australian Animal Health Laboratory, Geelong, VIC 3220, Australia

**Keywords:** Computational biology and bioinformatics, Genetics, Zoology

## Abstract

The Pama Croaker, *Otolithoides pama*, is an economically important fish species in Bangladesh. Intra-family similarities in morphology and typical barcode sequences of *cox1* create ambiguities in its identification. Therefore, morphology and the complete mitochondrial genome of *O. pama,* and comparative mitogenomics within the family Sciaenidae have been studied. Extracted genomic DNA was subjected to Illumina-based short read sequencing for *De-Novo* mitogenome assembly. The complete mitogenome of *O. pama* (Accession: OQ784575.1) was 16,513 bp, with strong AC biasness and strand asymmetry. Relative synonymous codon usage (RSCU) among 13 protein-coding genes (PCGs) of *O. pama* was also analyzed. The studied mitogenomes including *O. pama* exhibited consistent sizes and gene orders, except for the genus *Johnius* which possessed notably longer mitogenomes with unique gene rearrangements. Different genetic distance metrics across 30 species of Sciaenidae family demonstrated *12S rRNA* and the control region (CR) as the most conserved and variable regions, respectively, while most of the PCGs undergone a purifying selection. Different phylogenetic trees were congruent with one another, where *O. pama* was distinctly placed. This study would contribute to distinguishing closely related fish species of Sciaenidae family and can be instrumental in conserving the genetic diversity of *O. pama*.

## Introduction

Fishes are the primary animal protein source in Bangladesh. About 63% of protein demand is met by the fisheries sector^1^. Despite the country’s abundant sea resources, the marine sector contributes only about 14.9% to the total fish production^2^. Therefore, it is necessary to enhance marine fisheries resources and implement effective conservation programs for the economically important fish families. One such family is Sciaenidae, which comprises ray-finned fishes commonly known as drums or croakers due to their drumming noise production during spawning season^3^.

Family Sciaenidae includes a diverse group of marine and brackish-water fishes, consisting of approximately 311 species and 68 genera, distributed extensively across the Atlantic, Pacific, and Indian oceans^4,5^. Based on the most recent "Checklist of Marine Fish Species of Bangladesh," Bangladesh has 32 species of croakers belonging to 15 different genera^6^. The relative abundance of croakers in the Bay of Bengal (BoB) was found to be 12.8%, surpassing any other single group of marine fish^7^. Croakers constitute over 70% of the biomass distribution in the BoB. They are caught using both industrial and handmade trawlers, with an annual production of 41,943 metric tons in 2019–2020, accounting for 6.25% of the country's total marine catch^8^. These fishes are highly sought-after in both domestic and international markets. Around 86% of the total harvested croakers are dried and exported to various Asian countries, including Singapore, Japan, China and South Korea^9^. Among the different croaker fishes, the pama croaker *Otolothoides pama* stands out as one of the commercially important species in Bangladesh meeting 33.33% of total marine fish demand of Dhaka city^10^.

*Otolithoides pama* (Hamilton, 1822)^11^, also known as *Pama pama* (Fowler, 1933)^12^, is a benthopelagic carnivore that primarily feeds on shrimps and small fishes^13^. This migratory fish species is commonly found in estuaries and the BoB, with a seasonal migration to inland rivers, especially the Meghna River, during the monsoon for breeding purposes^14^. Although the IUCN Red List of Bangladesh (2000) does not list *O. pama* as a threatened species, its population has experienced a decline in recent years in its natural habitat, the BoB, primarily due to overfishing^9^. Despite its significance, research on *O. pama* has been relatively limited to its proximity analysis, reproductive pattern, growth pattern, otolith shape analysis, food and feeding habits, stock analysis, and barcoding of the *cox1* gene for species identification, especially in countries such as Bangladesh, India, and Myanmar^13,15–21^.

Precise identification is an essential prerequisite for the detailed study of an organism. The identification of *O. pama* based on morphology is critical since the members of the family Sciaenidae have a lot of ambiguity caused by morphological similarities and overlapping meristic counts among them. On the other hand, molecular tools have been extensively useful in fish species and fish-derived product identification^22^. As NCBI contains publicly sourced sequences, there is always a chance of misidentification, which may result in erroneous identification. Therefore, the present study has focused on the acquisition of substantial sequence data of mitogenome for unambiguous molecular identification of the species.

Metazoan mitochondrial DNA is a circular molecule of 15 to 20 kb length, comprising 37 genes, including two rRNAs, 22 tRNAs, 13 protein-coding genes, and a large non-coding control region (CR)^23–25^. It is distinguished by its small size compared to the nuclear genome, rapid evolutionary rate, high copy number, relatively conserved gene content and organization, rare genetic recombination, and maternal inheritance^26–28^. Mitogenomes have been widely employed to test microevolutionary theories and investigate phylogeography, population structures, and phylogenetic relationships among different organisms^27,29,30^. The mitogenome also offers substantial amount of information that can be used in resolving ambiguities among species and populations^31^. Hence, a reference genomic dataset for croaker fishes needs to be established for species enrichment, breeding technique enhancement, and aquaculture in future. The complete mitogenomes of 30 species of Sciaenidae other than *O. pama* were available in the NCBI GenBank database. Therefore, the main objective of the study was to sequence, assemble and annotate the complete mitogenome of *O. pama*. The assembled mitogenome was compared with that of other croakers from the same family. This comparison aimed to identify the most suitable mitochondrial DNA regions utilizable for removing taxonomic uncertainties, and monitoring population genetic status of the species.

## Results

### Identification based on morphometry and DNA barcode

**Figure 1:**
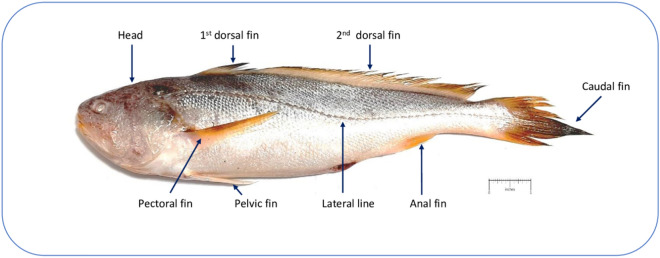
Morphological features of *Otolithoides pama.* The species shows light brownish along back and white beneath coloration. Fins are yellowish to pale orange. Length usually 50 cm in length with 1st, 2nd dorsal fins (D), pectoral (P_1_), pelvic (P_2_), anal (A) and rhomboid shaped caudal fin (C). Fin features are—D IX + I/41–45; P_1_ 17; P_2_ I/5; A II/7; C 17. Lateral line contains around 84 scales.

The collected specimen was identified as *O. pama* based on early literatures^14,32–34^. It had an elongated body with an oval head and a tapering posterior, round snout, circular eyes, and terminal mouth (Fig. 1). The scales were cycloid on the head and ctenoid on the body. The dorsal fin had a weak notch, and the spines were not very strong. The pectoral fins were pointed and as long as the head. The second, third and fourth spines of the dorsal fin were of equal size. The caudal fin was rhomboid. The lateral line continued up to the tip of the caudal. The body color was light brownish along the back and white beneath while the fins were yellowish^14,34^. Besides, the partial sequence of mitochondrial *cox1* gene (Accession: PP587739) was obtained from Sanger sequencing which is regarded as typical DNA barcode for fish species identification. The sequence was BLASTn-searched in NCBI GenBank database which showed 100% identity with *Argyrosomus thorpei* (Accession: MN259186.1, MK358927.1, MG969524.1, KY024210.1) and 99.67% identity with *Otolithoides biauritus* (Accession: MK340672.1) in addition to 99–100% identity with previously submitted *O. pama* sequences (See details in Table 1). These other two species belong to the same family as *O. pama*. All the *cox1* sequences originating from *O. pama* were 99% identical with the sequence of the present study. For in-depth investigation, all the *cox1* sequences of *A. thorpei* and *O. biauritus* were also retrieved and aligned to analyze sequence similarities. Interestingly, some *A. thorpei* sequence entries at NCBI showed 14% dissimilarity among themselves. Such an amount of within-species dissimilarity in the DNA barcode region is very unlikely to occur. The existing *cox1* sequences of *O. biauritus* also showed similar discrepancies among themselves. Further, identification of *O. pama* was confirmed by comparing its morphological characteristics, especially presence of the second dorsal fin (41–45 rays). with that of *A. thorpei* and *O. biauritus*^14,32–38^ (Table 1). Thus, the species could not be distinguished unambiguously based on the partial sequence of *cox1* gene.

**Table 1:** Morphological differences among *O. pama*, *A. thorpei* and *O. biauritus* and NCBI BLASTn search results of the sequenced *cox1* region of *O. pama.*

Features	*Otolithoides pama*	*Argyrosomus thorpei*	*Otolithoides biauritus*
Body coloration	Light brownish along back and white beneath. Fins are yellowish to pale orange^14,32,35^	Body silvery and greyish, fins are yellow or orange brown^36^	Dorsal greyish silvery with golden tinge, paler on belly. Fins yellowish to pale orange^33,35,37^
Fin features	D IX + I/41–45; P_1_ 17; P_2_ I/5; A II/7; C 17^14,32^	D1 XI, D_2_.I/26–28, A.II/6–7^36^	D IX + I/27–32; P_1_ 17; P_2_ I/5; A II/7; C 17^33,35,37,38^
Lateral line scales	84^14,34^	51–52^36^	50–60^35,37^
Size	± 50 cm usually, maximum 160 cm^14,33^	Usually 70 cm^36^	Usually 100 cm, max 160 cm^33^
Caudal fin	Rhomboid^14^ Pointed^32,33^	Truncate^36^, Rhomboid^34^	Pointed^33,35^
Location	Bay of Bengal, Burma, Malaysia, Eastern Indian Ocean^14,33^	Africa, Coastal region of Bangladesh^36,37^	Eastern Indian Ocean, Western Pacific Ocean, Philippines, Malaysia, Saint Martins, Bangladesh^33,37^
GenBank accession (cox1)	**MG787254.1, MH165298.1, MT815727.1, MT815726.1, MT815725.1, MT815724.1, MT815723.1, MN703111.1, OQ346271.1, MF611579.1, NC_080231.1**	**MN259186.1**, **MK358927.1**, **MG969524.1**, **KY024210.1**, KJ566675.1, KJ566676.1, KJ566677.1, KJ566678.1, KJ566679.1, JF492885.1, JF492886.1, JF492890.1, OM574579.1, JF492889.1	**MK340672.1**, MN512008.1, MF383188.1, MF383187.1, EF534127.1, EF536893.1, EF536890.1, JX983434.1, JX983435.1
Query coverage	100%, 97%, 96%	100%	100%
E value	0.0	0.0	0.0
Percent Identity	> 99% (all)	> 99% (bold font accessions only)	> 99% (bold font accessions only)

D_1_: first dorsal fin rays; D_2_: second dorsal fin rays; P_1_: pectoral fin rays; P_2_: pelvic fin rays; A: anal fin rays; C: caudal fin rays. Roman numerals: number of bony rays; Arabic numerals: number of soft rays.

Accession numbers in bold showed the highest sequence similarities (> 98%) with *O. pama cox1* partial sequence. The relevant references are added in square brackets.

### Features of *Otolithoidespama* mitogenome

**Figure 2:**
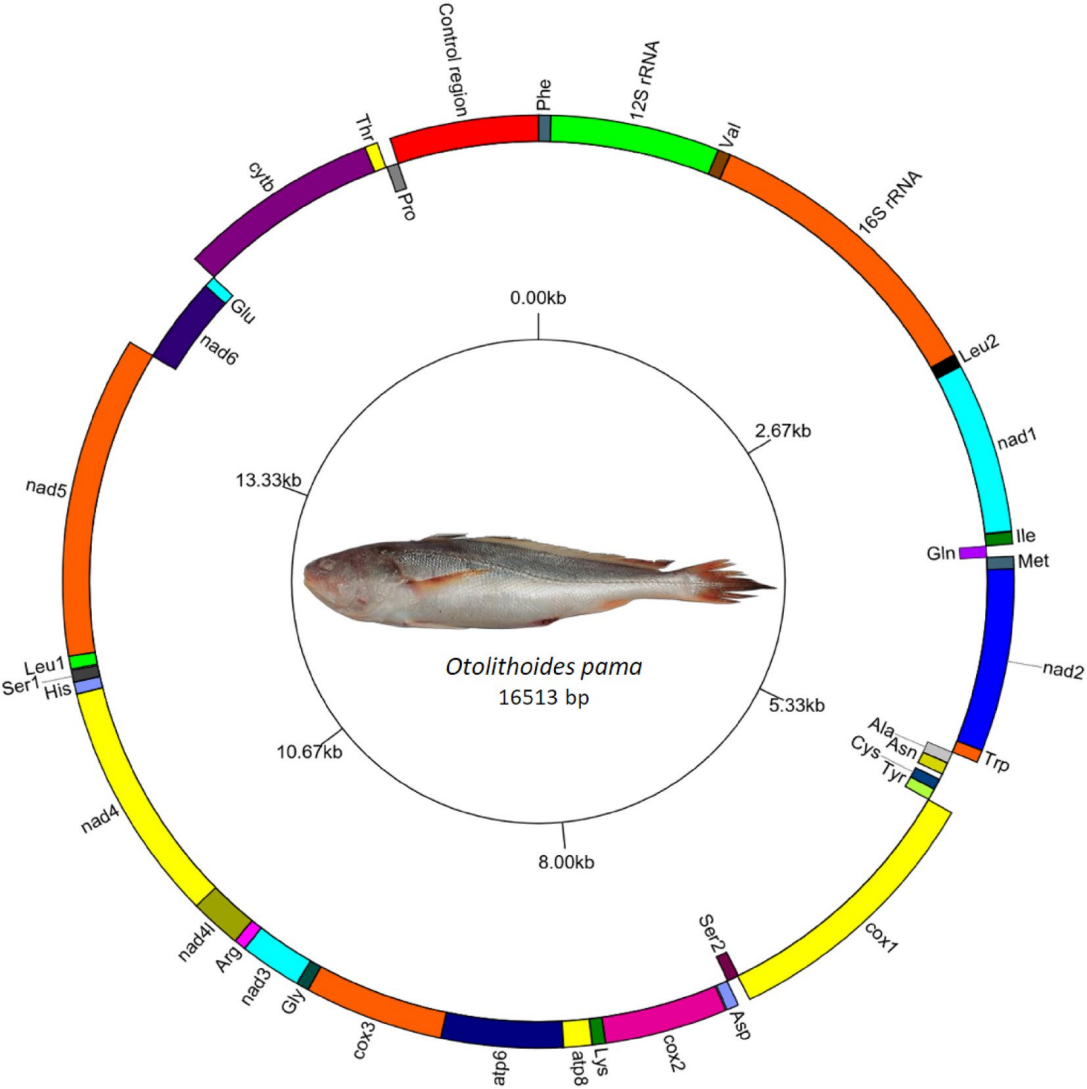
The complete mitochondrial genome of *O. pama*. The genes on the heavy strands are represented on the outer side of the circle and the genes on the light strand are on the inner side of the circle. The figure of the specimen used in this study is placed on the center of the circle.

The complete mitogenome of *O. pama* was assembled and annotated from NGS data. It was compact and 16,513 bp in length (NCBI Accession No: OQ784575.1) and contained all 37 genes found in typical teleost mitogenomes^39^, including two rRNA genes (*12S rRNA and 16S rRNA*), 13 protein-coding genes (*cox1-3, nad1-nad6, nad4l, atp8, atp6 and cytb*), 22 tRNA genes and two non-coding regions viz*.* O_L_ (Origin of light strand replication) and the CR. A circular genome map was constructed (Fig. 2). All tRNAs except for *tRNA*^*Ser*(AGY)^ formed a prominent cloverleaf-shaped putative secondary structure. Only *tRNA*^*Ser(AGY)*^ could not be folded into a clover-leaf structure because of lacking the DHU-arm (Supplementary Fig. S1).

Protein Coding Genes (PCGs) ranged from 168 bp (*atp8*) to 1839 bp (*nad5*) with a total length of 11,437 bp including four overlaps of 22 bp between seven PCGs (Table 2). The PCGs comprised 69.26% of the complete mitogenome. All the PCGs started with ATG start codon as typical vertebrate mitogenome. A wide diversity in termination codon was observed. Nine PCGs had complete termination codons. Of them, seven had TAA as stop codon, whereas *cox1* and *nad1* had AGA and TAG, respectively. Moreover, incomplete termination codon T– was found in four PCGs (*cox2, nad3, nad4* and *cytb*) and the post-transcriptional polyadenylation is thought to have completed these codons as TAA^29^.

**Table 2:** Genomic features of the complete mitochondrial genome of *O. pama*.

Gene	Position		Intergenic nucleotide (bp)	Nucleotide length (bp)	Codon		Amino acid length (aa)	Anti-codon	Strand
	Start	End			Start	Stop			
*tRNA*^*Phe*^* (F)*	1	69	0	69				GAA	H
*12S rRNA*	70	1019	0	950					H
*tRNA*^*Val*^* (V)*	1020	1091	1	72				TAC	H
*16S rRNA*	1093	2795	0	1703					H
*tRNA*^*Leu2*^* (UUR)*	2796	2869	0	74				TAA	H
*nad1*	2870	3844	4	975	ATG	TAG	324		H
*tRNA*^*Ile*^* (I)*	3849	3918	− 1	70				GAT	H
*tRNA*^*Gln*^* (Q)*	3918	3988	− 1	71				TTG	L
*tRNA*^*Met*^* (M)*	3988	4056	0	69				CAT	H
*nad2*	4057	5103	− 1	1047	ATG	TAA	348		H
*tRNA*^*Trp*^* (W)*	5103	5174	0	72				TCA	H
*tRNA*^*Ala*^* (A)*	5175	5243	2	69				TGC	L
*tRNA*^*Asn*^* (N)*	5246	5318	0	73				GTT	L
O_L_	5319	5353	0	35					L
*tRNA*^*Cys*^* (C)*	5354	5419	0	66				GCA	L
*tRNA*^*Tyr*^* (Y)*	5420	5489	1	70				GTA	L
*cox1*	5491	7047	− 5	1557	ATG	AGA	518		H
*tRNA*^*Ser2*^* (UCN)*	7043	7113	3	71				TGA	L
*tRNA*^*Asp*^* (D)*	7117	7185	7	69				GTC	H
*cox2*	7193	7883	0	691	ATG	T–	230		H
*tRNA*^*Lys*^* (K)*	7884	7957	1	74				TTT	H
*atp8*	7959	8126	− 10	168	ATG	TAA	55		H
*atp6*	8117	8800	− 1	684	ATG	TAA	227		H
*cox3*	8800	9585	− 1	786	ATG	TAA	261		H
*tRNA*^*Gly*^* (G)*	9585	9655	0	71				TCC	H
*nad3*	9656	10,004	0	349	ATG	T–	116		H
*tRNA*^*Arg*^* (R)*	10,005	10,073	0	69				TCG	H
*nad4l*	10,074	10,370	− 7	297	ATG	TAA	98		H
*nad4*	10,364	11,744	0	1381	ATG	T–	460		H
*tRNA*^*His*^* (H)*	11,745	11,813	0	69				GTG	H
*tRNA*^*Ser1*^* (AGY)*	11,814	11,881	7	68				GCT	H
*tRNA*^*Leu1*^* (CUN)*	11,889	11,961	0	73				TAG	H
*nad5*	11,962	13,800	− 4	1839	ATG	TAA	612		H
*nad6*	13,797	14,318	0	522	ATG	TAA	173		L
*tRNA*^*Glu*^* (E)*	14,319	14,387	4	69				TTC	L
*cytb*	14,392	15,532	0	1141	ATG	T–	380		H
tRNA^Thr^ (T)	15,533	15,604	4	72				TGT	H
*tRNA*^*Pro*^* (P)*	15,609	15,678	0	70				TGG	L
Control region (CR)	15,679	16,513	0	835					H

The table contains start and end positions of each genes, intergenic nucleotide size, gene size, start and stop codons and Amino acid length of Protein coding genes, anticodon info for non-protein coding genes and strand information. For intergenic nucleotide, positive and negative values indicate overlap and gaps between adjacent genes, respectively. In strand column, H and L denote the genes are encoded on the heavy and light strands, respectively.

**Figure 3:**
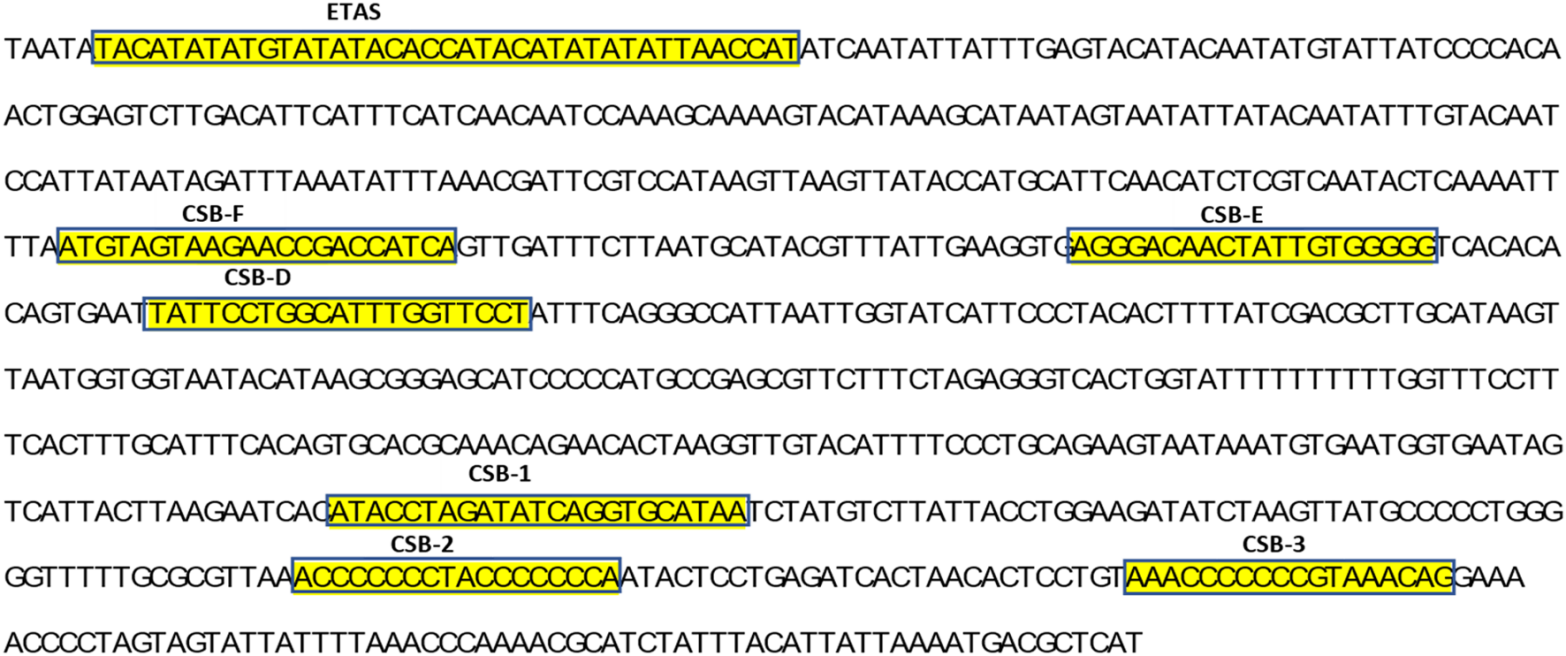
The partial sequence of the control region showing the conserved blocks (ETAS, CSB-F, CSB-E, CSB-D, CSB-1, CSB-2, and CSB-3) in highlighting colors and boxes.

The non-coding O_L_ region, positioned between *tRNA*^*Asn*^ and *tRNA*^*Cys*^, was 35 bp long and assumed to be significant in relation to the origin of light strand replication^40^. The CR was 835 bp, located between *tRNA*^*Pro*^ and *tRNA*^*Phe*^. It was flanked by a stretch of TAATATA at the 5'-end. In the CR, an extended termination-associated sequence (ETAS), central conserved region (CSB-D, CSB-E and CSB-F) and three conserved blocks (CSB1, CSB2, and CSB3) were detected (Fig. 3). These domains were detected based on previous studies on the mitogenome of *Miichthys miiuy*^41^ and *Larimichthys crocea*^42^*.* The segment CSB-1 varied within these three species except for the 2 bp start and 8 bp end sequences. The GTGGG-box which is a typical feature of the conserved domain CSB-E in teleosts were also found in *O. pama* CR^42^.

In the mitogenome, a total of 10 intergenic spacer regions (IGS) ranging from 1 -7 bp and nine overlapping regions ranging from 1–10 bp were found among the genes which were typical of most teleosts^39,43,44^.

### Nucleotide composition and skewness

The mitogenome of *O. pama* was comprised of 29.63% A (4,892), 26.18% T (4,323), 14.64% G (2,417), and 29.56% C (4,881). The overall A + T content of the mitogenome was higher (55.81%) than the G + C content (44.20%) (see details in Supplementary Table S1). Total A + T content of the PCGs accounted for 55.60%, with the lowest (50.17%) in *nad4l* and the highest (67.26%) in *atp8*. Among 22 tRNAs, A + T content was the lowest (43.06%) in *tRNA*^*Thr*^ and the highest (69.01%) in *tRNA*^*Gly*^*.* Average A + T content in two rRNAs, 22 tRNAs, and 13 PCGs were 53.85%, 55.92%, and 55.96%, respectively. The highest A + T content (64.20%) was observed in the CR (Supplementary Table S1).

The overall AT-skew and GC-skew of the mitogenome were positive (0.061) and negative (− 0.337), respectively (Supplementary Fig. S2). The AT and GC-skew values of the 13 PCGs were − 0.025 and − 0.362, respectively, indicating a clear TC bias in the PCGs which is consistent when compared to others in the family. Most of the PCGs (8 PCGs) showed a negative AT-skew and all the PCGs showed a negative GC-skew except for *nad6* which showed a positive GC-skew that was coherent to other 16 species of the family^4^.

### Relative synonymous codon usage (RSCU)

**Figure 4:**
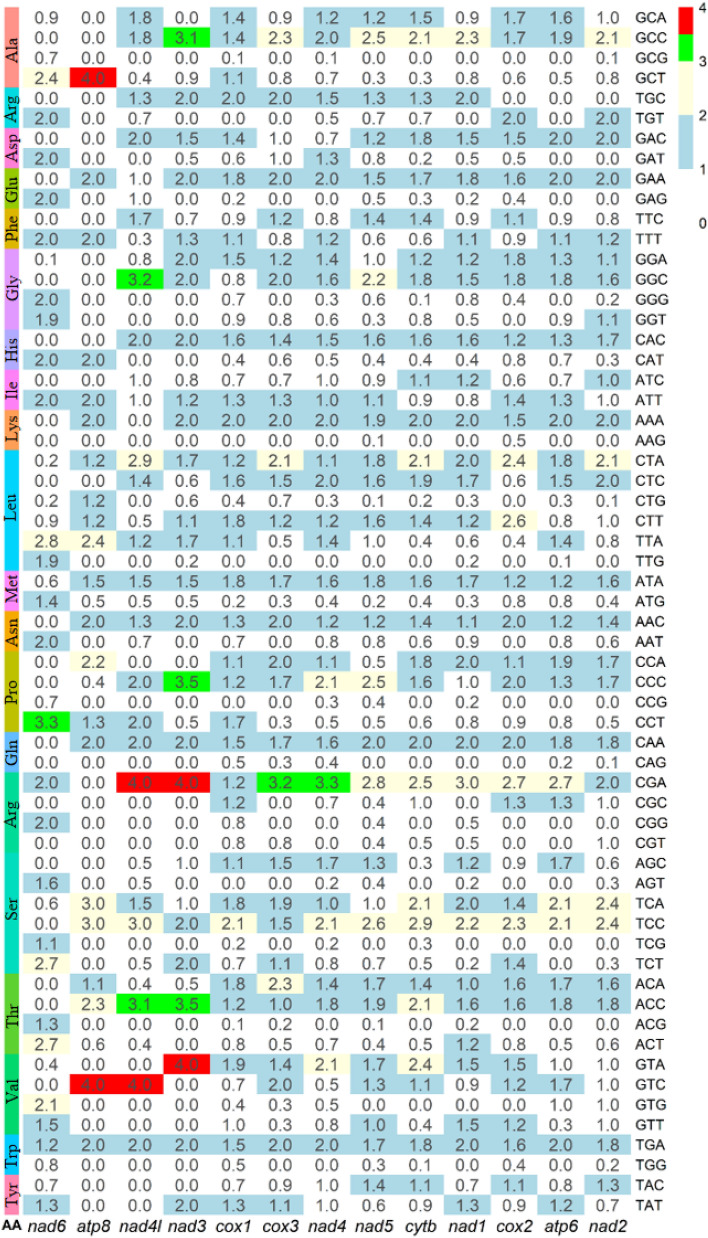
Relative synonymous codon usage (RSCU) of the 13 protein coding genes of *O. pama.*

Across the 13 PCGs, the amino acids Leucine (Leu) and Serine (Ser) were most frequent having usages of 6 different codons (10% each). Alanine (Ala), Glycine (Gly), Proline (Pro), Arginine (Arg), Threonine (the) and Valine (val) used 4 different codons (6.67% each) (Fig. 4). The rest of the amino acids used only two codons (3.33% each).

Among the 60 codons, 11 codons (GCT of Alanine, ATT of Isoleucine, TTA, CTT and CTA of leucine, ATG and ATA of Methionine, TTT of Phenylalanine, CCT of Proline, TCA of Serine and TGA of Tryptophan) appeared across all the 13 PCGs (RSCU > 0). The TGA codon of Tryptophan showed RSCU > 1 value across all 13 PCGs, which showed the highest abundance of this codon across all PCGs. Besides, while we sum up the RSCU values across the PCGs, CGA of Arginine got higher cumulative RSCU values (33.31) than the TGA of Tryptophan (23.71). On the other hand, low RSCU values (RSCU < 1) has been observed in 5 codons (TGG of Tryptophan, CAG of Glutamine, GCG of Alanine, CCG of Proline and AAG of Lysine), where RSCU < 1 appeared across 12 PCGs.

### Comparative genomics among 31 Sciaenid species

**Figure 5:**
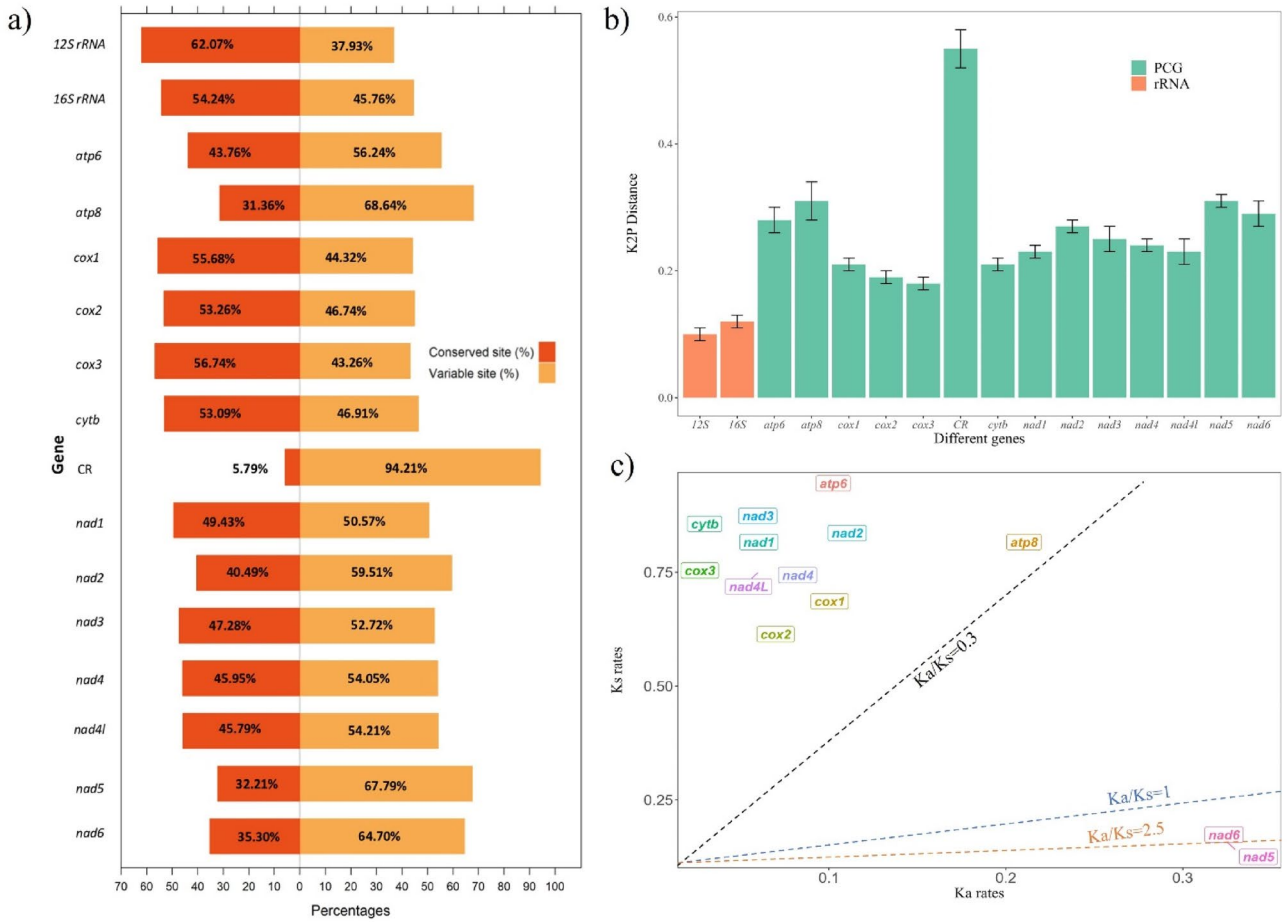
Comparative genomics across the family Sciaenidae. (**a**) conserved and variable regions (**b**) overall K2P distances and (**c**) Ka/Ks substitution of 13 PCGs.

Comparative genomics among the 31 species of Sciaenidae family (Supplementary Table S2) was carried out to understand the basic and unique properties of their mitogenomes, shared features and differences to elucidate their impact on family relationship and taxonomic positions. This study included the conserved-variable site ratio, K2P distance (Kimura 2 parameter model for estimating genetic distance), Ka/Ks substitution ratio (the rate of non-synonymous substitution, Ka to synonymous substitution, Ks) of 13 PCGs (Fig. 5), and phylogenetic studies.

The percentages of conserved and variable sites revealed that *12S rRNA* was the most conserved gene (62.07% conserved) and CR was the least conserved region (only 5.79% conserved). Among all PCGs, *cox3* was the most conserved gene (56.74%) and *atp8* was the least conserved gene (31.36%), which was vice-versa for variance (Fig. 5a). The percentage of variable site of CR region was astonishingly high (94.2%), making it the hypervariable region of the mitogenome.

Intra-family K2P genetic distances were estimated for 31 species of the family. K2P distances of two rRNAs, 13 PCGs, and the CR region of the family Sciaenidae revealed that *12S rRNA* was the least distant (0.1 $\pm 0.01$) among all and the CR the most distant (0.55 $\pm$ 0.03) (Fig. 5b). Both the rRNAs showed relatively lower distance compared to other genes. Among PCGs, *cox3* was the least distant (0.18 $\pm 0.01$) and both *atp8* and *nad5* were the most distant (0.31 $\pm 0.03$ and 0.31 $\pm 0.01$, respectively). Hence, *nad5* and *atp8* most likely had the sciaenids' most rapid evolutionary rate, while *cox3* had the slowest.

### Selective pressure analysis

The "Ka/Ks" ratio estimates the rates of non-synonymous and synonymous substitutions in the coding sequences. The Ka/Ks ratio was the lowest (0.0393) in *cox3* indicating the gene had undergone lowest rate of evolution and the highest (2.5161) in *nad5* indicating its highest evolution rate (Fig. 5c). The Ka/Ks values for two PCGs, *nad5* and *nad6*, where substitution rates were 2.5161 and 2.4346, respectively (Ka/Ks > 1), indicated positive selection during evolution. The Ka/Ks ratio for the rest of the PCGs were less than 1.

### Intra-family gene rearrangement

The mitochondrial genome arrangement of *O. pama* was compared with 30 other species of Sciaenidae available in the NCBI Genbank (Supplementary Table S2).

**Figure 6:**
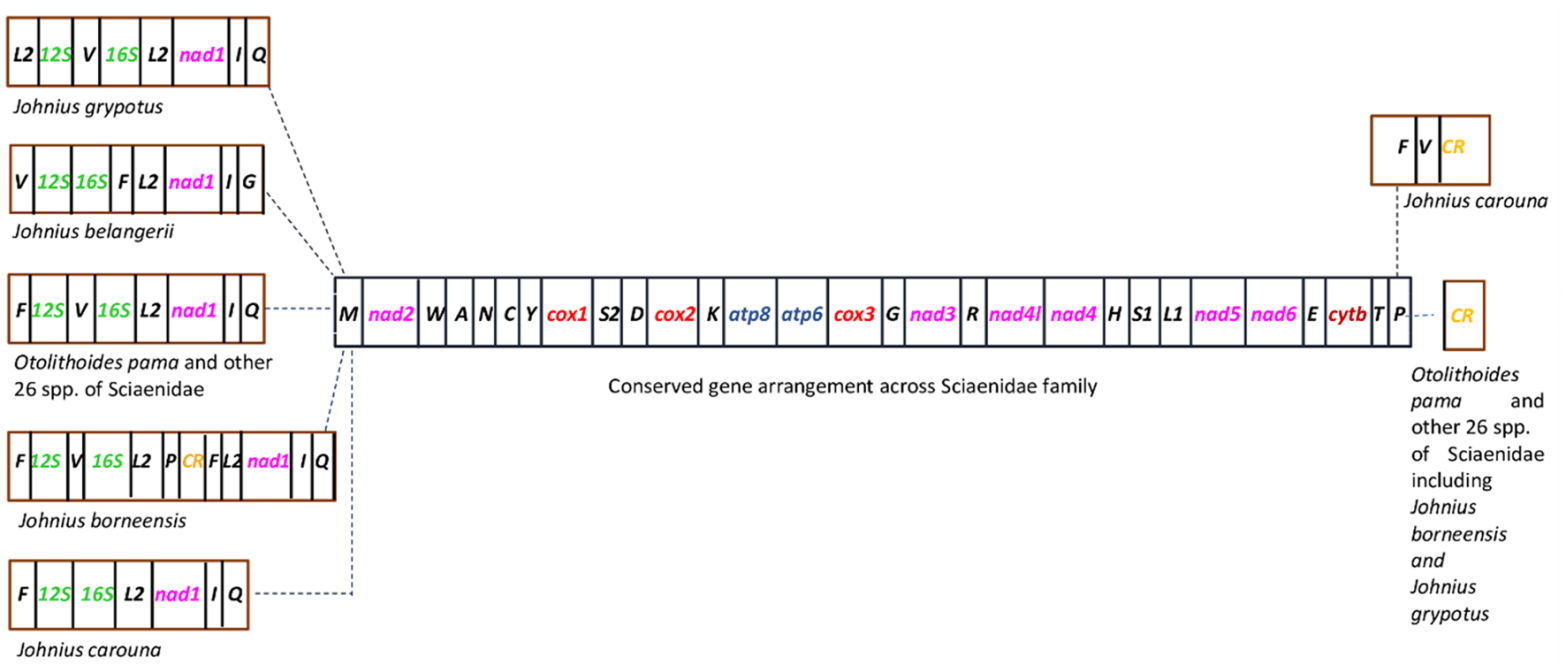
Gene arrangement of mitochondrial genes within 31 species of Sciaenidae family.

The mitogenome size and gene order were consistent across the family except for the genus *Johnius* (Fig. 6). While excluding *Johnius* group, the size ranged from 16,408 bp (*Pennahia pawak*) to 16,842 bp (*Atrobucca nibe*) as shown in Supplementary Table S2. The genus *Johnius* possessed exceptionally longer mitogenomes; which were 19,154 bp, 18,630 bp, 18,752 bp, 18,523 bp for *J. belangerii, J. borneensis, J. carouna,* and *J. grypotus*, respectively. Overall, mitogenome size, arrangement and cluster distribution of *O. pama* and most species of Sciaenidae (excluding the *Johnius* spp.) were canonical and consistent with other teleost fishes^45–47^.

The CR was absent in *J. belangerii* and there was duplication of CR in *J. borneensis. J. borneensis* also contained an extra copy of *tRNA*^*Phe*^*, tRNA*^*Leu(UUR)*^*, and tRNA*^*Pro*^ each. *J. grypotus* had an extra copy of *tRNA*^*Le*u(UUR)^ while lacking *tRNA*^*Phe*^. According to Xu et al.^48^, the CR of *J. belangerii*, was substituted by a stretch of AT-rich non-coding sequence between *tRNA*^*Pro*^ and *tRNA*^*Val*^^48^. *Johnius* was the earliest genus to be evolved within the family according to a phylogenetic analysis and could be a prove of relatively slower rate of change in Sciaenidae over time^48^. However, an alternate hypothesis, discussed in Wen et al.^49^, posits that this genus may be of recent origin. Incorporating nuclear genomic data could be instrumental in accurately establishing its position.

### Phylogenetic analysis

**Figure 7:**
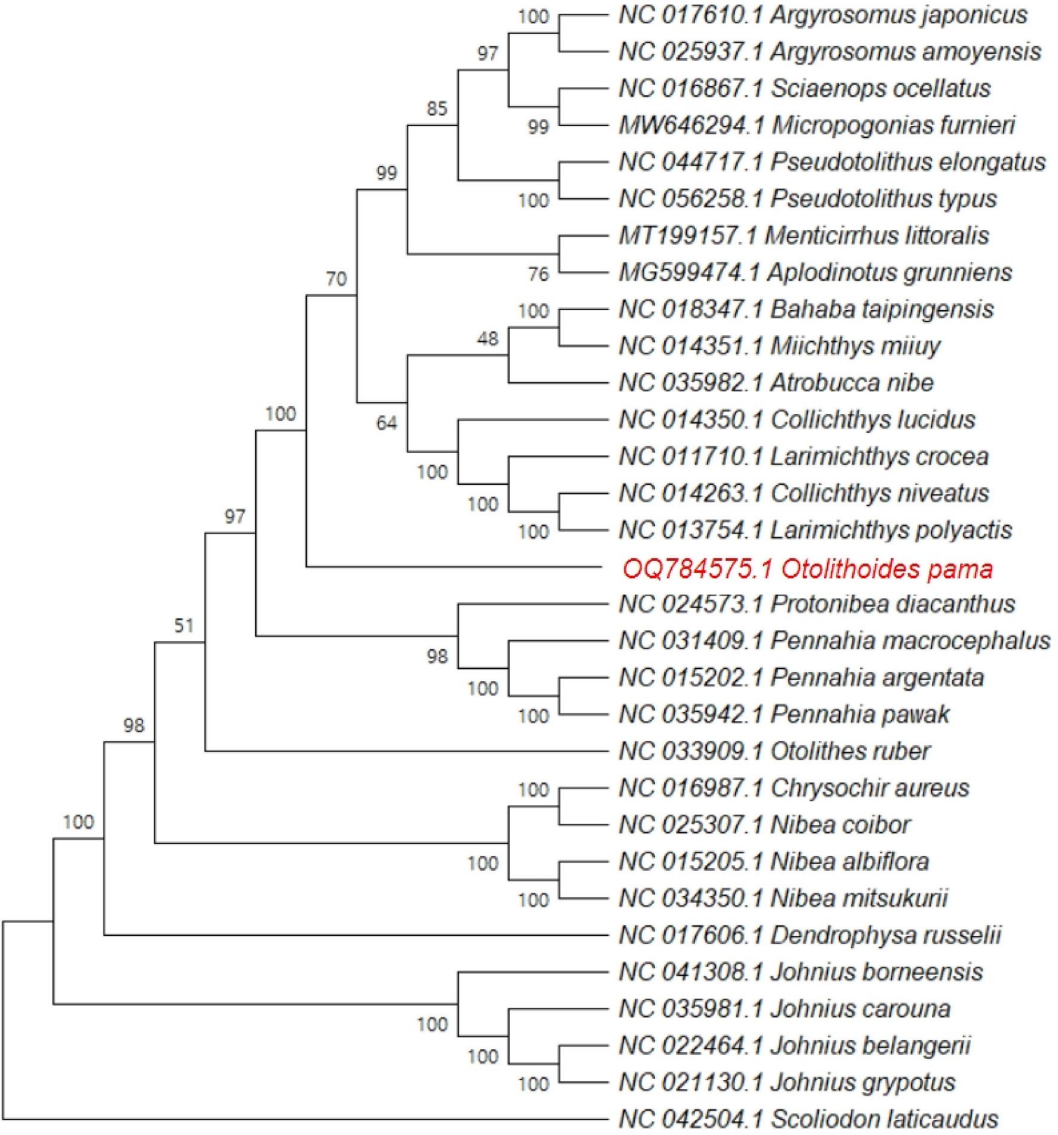
The phylogenetic tree of the family Sciaenidae, based on complete mitochondrial genomes. The tree was constructed by the Maximum-Likelihood (ML) method with 1000 bootstrap replicates and *Scoliodon laticaudus* was used as an outgroup. The bootstrap values (posterior probabilities) are shown on the nodes. The GenBank accession numbers precede the respective species names.

A phylogenetic tree was generated to find the position of *O. pama* among 30 other sciaenids based on complete mitochondrial genomes (Fig. 7). *Scoliodon laticaudus* (Family: Carcharhinidae) was used as an outgroup. Regardless of the analytic method utilized, the phylogenetic trees were highly synchronous with high posterior likelihoods and bootstrap values. In the phylogenetic tree, four *Johnius* species formed a separate sister clade due to their mitogenome sequence peculiarities and *Dendrophysa russelli* is their close relative^50^.

The *Collichthys*, *Larimichthys*, *Atrobucca*, *Miichthys*, and *Bahaba* genera were clustered together as a clade indicating their close evolutionary relationship. The species of *Collichthys*, *Larimichthys*, *Atrobucca*, and *Miichthys* genera were placed together as a separate subfamily Pseudosciaeniae based on morphological characteristics^41^. Another phylogenetic analysis revealed that *Bahaba taipingensis* is more strongly linked to Pseudosciaeniae. *Miichthys miiuy* is its sister taxon, and the two are related to *Collichthys* and *Larimichthys*^51^. This result was consistent with previous findings^41,50^. *Argyrosomus* genus formed a clade by clustering with sister taxon *Sciaenops ocellatus* and *Micropogonias furnieri* with highest branch support which was seen in previous phylogenetic study^52,53^. They all seemed to be close relatives of *Pseudotolithus* genus, *Aplodinotus grunniens*, and *Menticirrhus littoralis*^50,54^. *Protonibea diacanthus* formed a separate clade with the genus *Pennahia*. The genus *Nibea* formed a sister clade with *Chrysochir aureus* which was further related to *Otolithes ruber*. Similar results were reported previously^50,54^.

The phylogenetic analysis revealed that our present study *O. pama* was positioned among the other sciaenids and was closely related to *Collichthys*-*Larimichthys* and *Pennahia-Protonibea* sister group with high branch support value. The result was reliable and clear to elucidate its phylogenetic position within the family. The outgroup species *S. laticaudus* formed a distinct branch from the other 30 croakers indicating that the species is evolutionary distant from the family and became separated from their common ancestor long time ago.

The phylogenetic tree based on the PCGs and average RSCU (Supplementary Figs. S3 and S4) closely resembled the tree constructed from the complete mitogenomes regarding the placement of *O. pama*. In the hierarchy tree based on RSCU values, the position of *O. pama* remained consistent across three out of the four categories of trees (Complete, Average, Ward, and Single). In these trees, there are examples of different species clustering together and species of the same genus clustering separately, but *O. pama* was distinct.

Nonetheless, it is worth highlighting that even the genus *Argyrosomus* consistently formed a distinct cluster that was distant from *O. pama* in all the phylogenetic trees constructed from the complete mitogenome, PCGs and average RSCU; further emphasizing the distinctiveness of *O. pama* within the family Sciaenidae.

## Discussion

The Sciaenidae family was previously categorized as Perciformes, but more recent research has revealed that it evolved earlier from the Perciformes with significant evolutionary distance^4,5^. The family was moved into a complex series Eupercaria, there are still many questions regarding the phylogeny of this family^4^. Consequently, proper identification of species of this family is problematic because of the morphological ambiguities among closely resembled species. The partial sequence (599 bp) of *cox1* gene of *Otolithoides pama* (Accession: PP587739) was unable to identify the species unambiguously as the BLASTn search resulted in more than 99% identity with other relative species such as *A. thorpei* and *O. biauritus*. This could have resulted from either true similarity of sequences in the targeted mitochondrial *cox1* region or occurrence of possible hybrids as mentioned elsewhere or misidentification of the species^55^. Analysis of the available sequences (n = 14) of *A. thorpei* showed highest 14.41% (out of 562 bp) intra-species dissimilarity (between KJ566675.1 and KY024210.1) in the *cox1* gene region which is too high to have in the same species. One of the available sequences of *O. biauritus* also showed similar discrepancy. Thus, it is not impossible that *O. pama* was misidentified in some previous NCBI submissions (Accession: MN259186, MK358927, MG969524, KY024210, MK340672) as another species. A misinterpreted submission to NCBI database might easily result in additional similar entries because it may appear as the top hit in a BLASTn search.. In the present study, *O. pama* was rigorously identified using distinct morphological characteristics, including dorsal fin rays and number of lateral line scales (Table 1). Further, to resolve taxonomic uncertainties, the complete mitogenome of *O. pama* has been sequenced. The full *cox1* sequence of *O. pama* could not be compared with that of the other two species due to their unavailability. Additionally, when the complete mitogenome of *O. pama* was BLASTn searched, it showed less than 90% similarity with other species. Notably, the available complete mitogenomes of two species of the genus *Argyrosomus* (*A. amoyensis* and *A. japonicus*) showed only 85% sequence similarity with *O. pama*, which is indicative of distinction between *O. pama* and the genus *Argyrosomus*. However, like *O. pama*, in-depth morphological and molecular studies of *A. thorpei* and *O. biauritus* would be invaluable.

The mitogenome of *O. pama* was successfully sequenced, assembled, and annotated for the first time (GenBank Accession: OQ784575.1). Its genome size and organization was typical to other teleosts^39^. The 13 PCGs comprised 69.26% of the mitogenome. All the PCGs started with “ATG” initiation codon which is a typical start codon in teleost mitogenome. The structures and anticodons of 22 tRNAs were highly conserved across the family. All the tRNAs folded into a cloverleaf-like structure except for *tRNA*^*Ser1(AGY)*^ that lacked a DHU-arm which is a common feature of vertebrate mitogenome^41,43,56,57^. In *O. pama* mitogenome, two long IGS (7 bp each) and two long overlaps were detected. The largest overlapping sequence “ATGACTGTAA” was located between *atp8-atp6* and other 7 bp overlapping sequence “ATGCTAA” was located between *nad4l-nad4* which was conserved among vertebrates^58–60^. The CR of Sciaenidae family exhibited significant length variation. In the CR region of *O. pama*, ETAS, CSB-D, CSB-E, CSB-F, CSB-2, and CSB-3 domains were detected following previous reports on *Miichthys miiuy*^41^ and *Larimichthys crocea*^42^*.* ETAS region is hypervariable and can be useful for analyzing interspecific variations^41^. CSB-D is highly conserved in teleosts and may control H-strand replication, initiate the CR structure, and possibly functions in mitochondrial metabolism^61,62^.

Most amino acids can be transcribed by numerous synonymous codons because of the degeneracy of the genetic code^63,64^. Different species spontaneously produce synonymous codons at different rates. Protein expression, structure, and function may be impacted by the choice of codons used^63^. If there is no codon usage bias, the RSCU value is 1.0. Codons that are used more rarely than anticipated will have RSCU values below 1.0, whereas those that are utilized more often than anticipated would have RSCU values over 1.0. In the PCG’s of *O. pama* mitogenome, Leu and Ser amino acids were utilized by six different codons, while all other amino acids were encoded by either two or four which was consistent with other teleosts^4,65^. In PCGs, codon usage is crucial in regulating gene expression levels and is influenced by translational selection^66,67^. This selection is for translational efficiency, accuracy and protein synthesis, where different organisms opt for codons that can be quickly processed to reduce the duration and energy spent on translation^66,68–70^. Translational selection occurs in highly expressed genes, which have a higher frequency of preferred codons than poorly expressed genes^66,67^.

Analysis of the conserved and variable sites of the 31 sciaenid mitogenomes revealed that *12S rRNA* and CR were the most conserved and variable mitochondrial DNA regions, respectively. The multiple sequence alignment of 22 tRNAs revealed that they were highly conserved across the family. The K2P model^71^ is used to calculate the amount of genetic variation between two nucleotide sequences based on nucleotide changes that have taken place during the course of evolution^72^. When genetic distances are minimal, the K2P distance model is the most useful^73^. The K2P genetic distance of two rRNAs, 13 PCGs and the CR of 31 species of the family again demonstrated that *12S rRNA* is the most conserved and CR is the most variable region. Among PCGs, *cox3* had the lowest K2P genetic distance and the highest conserved site ratio corresponding to a previous report on sciaenids^4^. Another *cox* gene, *cox1* was reported to be the most conserved mitochondrial PCGs among 250 fishes including both ray-finned and cartilaginous fishes^74^. This variation in the results could be attributed to dissimilarity in the number and groups of species included in the studies or might indicate distinctiveness of the sciaenids.

Homologous PCGs from closely linked species were compared by Ka/Ks ratio analysis. The pace of evolution between these two sequences is represented by the Ka/Ks ratio, which is calculated by dividing the number of non-synonymous (amino acid) substitutions per non-synonymous site (Ka) by the number of synonymous substitutions per synonymous site (Ks). This ratio also shows the pressure of selection on organism’s evolution. The Ka/Ks ratio of 13 PCGs were calculated for 31 species of Sciaenidae, where the ratio ranged from 0.0393 (*cox3*) to 2.5161 (*nad5*), meaning that *cox3* had undergone the lowest rate of mutation and *nad5* had undergone the highest rate of mutation. This finding is consistent with the previous results of K2P genetic distance and conserved site ratio, further strengthening their evolutionary correlation. A similar trend of lowest Ka/Ks ratio in *cox3* and highest Ka/Ks ratio in *nad5* has been observed in three individual species of the same family, namely *N. coibor*, *P. diacanthus*, and *A. amoyensis*^4^. In general, the lowest and highest Ka/Ks ratios in *cox* and *nad* genes, respectively, were reported in other fishes (Cobioninae and Cyprinidae) and other organisms such as bugs, crayfishes, and testudines^75–80^. Interestingly, *nad5* and *nad6* genes of *O. pama* exhibited a Ka/Ks value greater than 1, indicating that they might have undergone positive selection where non-synonymous substitutions were prevalent over synonymous substitutions. Whereas in most other fishes, all the PCGs exhibited Ka/Ks value less than one including *nad5* and *nad6*^76,77^. The remarkable findings within the Sciaenidae family suggest that examining the link between *nad* genes and adaptation to different environments and ecological niches could provide valuable insights. The other PCGs had Ka/Ks < 1 values, indicating that the majority of the PCGs might have undergone purifying (negative) selection where synonymous substitutions were prevalent over non-synonymous substitutions. It demonstrated that mitochondrial PCGs were conserved during the evolutionary process, a pattern which has been reported in the mitogenome of *Cirrhinus reba*, (Family: Cyprinidae) and three sciaenids namely *N. coibor*, *P. diacanthus*, and *A. amoyensis*^4,65^. Mitochondrial PCGs play crucial role in cellular metabolism, ATP production and nucleotide biosynthesis which subjects mitogenomes under functional constraints and purifying selection^81^. This analysis showed that selection pressures varied on different genes and indicated how they evolved in different ways to ensure fundamental biological function and survival of *O. pama*.

A comparison of the genome organization and size of the members of Sciaenidae exhibited gene rearrangement in a group of species belonging to the genus *Johnius* (Accession: NC_022464.1, NC_041308.1, NC_035981.1, and NC_021130.1) which showed deletion of CR and *tRNA*^*Phe*^ and duplication of CR, *tRNA*^*Leu(UUR)*^*, tRNA*^*Phe*^, and *tRNA*^*Pro*^; a phenomenon previously reported^48^. These rearrangements were likely to be caused by CR sequence substitution, tandem duplication, transpositions, and shuffling of genes; all of which have been previously observed to be the principle causes of mitochondrial gene rearrangement in teleosts^57,74,82–85^. All the other species of this family including *O. pama* had a typical conservative mitogenome length and organization.

Complete mitochondrial genomes are often utilized in phylogenetic studies because they provide minor, consistent changes throughout time for any taxon. In this approach, all the mitochondrial genes together can represent phylogenetic information more effectively than a single nuclear or mitochondrial gene^31,46^. Although a few mitogenomic regions can be selected for easy identification or assessing population genetic status of the species, either *12S rRNA* or commonly used *cox1* regions alone may not be the appropriate for species identification of the Sciaenidae family. Besides *cox1* gene, we suggest to considering other moderately variable regions, i.e. *16S rRNA*, *cox2*, and *cytb*. For population genetics study, the target sequence can be selected from the most variable regions such as CR*, atp8*, *nad5*, *nad6*, and *nad2*.

Due to the small number of taxa sampled, the phylogenetic relationships among Sciaenidae members should be accepted with caution. Nevertheless, in a detailed morphology-based study of sciaenid, the family was divided into four branches, A, B, C and D, with sub-branches^86^. Most of the species of the present study belonged to the sub-branch D7 of the previous report, where the genus *Otolithoides* alone was placed in a distinct sub-branch D5^86^. Here, the distinct position of *Otolithoides* in the mitogenome-based phylogenetic tree corresponds with the morphology-based phylogeny. *Johnius*, *Nibea*, and *Dendrophysa*, which are relatively closer in the present phylogenetic tree, showed similar relationship within the D7 sub-branch of the morphological cladogram of Sasaki el al.^86^. The distance between *Sciaenops* and *Otolithoides* also corresponds to their placement in different branches C and D respectively in morphology-based cladogram^86^. To comprehend the links among the key lineages within the Sciaenidae, rigorous molecular and morphological characterization of more croakers, especially *A. thorpei* and *O. biauritus*, should be carried out. Since partial sequence of *cox1* gene cannot be universal in terms of barcoding and fish species identification, additional mitogenomic variable regions needed to be tested. Whole genome sequencing is encouraged to find out more important factors regarding the survival mechanism, fertility, morphological variation, and behavioral pattern of the species. Identification of economically important genes can be another significant factor to consider.

## Conclusion

Current study explored the complete mitogenome of *O. pama* and indicated its taxonomic position in relation to 30 other sciaenid fishes. The study resolved the ambiguity arising from *cox1* based identification, showed unique genetic fingerprints within the family—including positive selection of *nad5* and *nad6* genes, and finally comprehensive genomic analysis for considering additional mitochondrial regions for species identification and population genetics studies. As the study is limited to mitochondrial genome, future work on nuclear genome wide studies has potential to confidently resolve taxonomic ambiguities of the fish family. Current study can assist other applications such as fish product identification in trade monitoring, fisheries resource management plan etc.

## Methods

### Sample collection and identification

A fresh, dead specimen of *O. pama* was collected from a fish market near the Meghna River, Bangladesh (on 27 December 2022) and transported in an ice box to the Genetics and Molecular Biology Laboratory of Department of Zoology at University of Dhaka. The specimen was morphologically identified by following previous literatures^14,32–38^. For subsequent analysis, the sample was preserved in -20**°**C. Soon, genomic DNA was extracted from soft tissue of the dorsal fin base using CTAB method^87^. The extracted DNA was separated using 1.0% agarose gel electrophoresis and a region of the *cox1* gene was PCR-amplified and sequenced to identify the sample at the molecular level. The set of primers used for *cox1* amplification was, F primer: 5'-TCAACCAACCACAAAGACATTGGCAC-3', R primer: 5'-TAGACTTCTGGGTGGCCAAAGAATCA-3', with an amplicon length of 655 bp^88–90^. PCR was performed using a Thermal cycler (Model 0005.400; Creacon Technologies, Netherlands) and the paired-end Sanger sequencing of the amplified gene was carried out by 3500 Dx Genetic Analyzer (Applied Biosystems, ThermoFisher Scientific, New York, USA). The raw sequence from Sanger was viewed as chromatogram in FinchTV version 1.4.0^91^ where the quality of the chromatogram was checked. The sequences of both forward and reverse reads (reverse complemented) were aligned in Serial Cloner version 2.6.0^92^ to find the matching regions and edited finally to have a quality sequence. The partial sequence of *cox1* gene (Accession: PP587739) was subjected to BLASTn search against NCBI standard nucleotide collection database to find highly similar sequences.

### Illumina short-read sequencing and processing

The highly concentrated extracted DNA was purified using PCR clean-up spin protocol (Axygen^®^ AxyPrep Mag PCR Clean-Up Kit, Corning Life Sciences, USA). The quality and quantity of the DNA was estimated by NanoDrop Spectrophotometer (Thermofisher Scientific^TM^, USA). Then, the high-quality DNA was sent to Azenta Life Sciences (Burlington, MA 01,803, USA) for sequencing. During the sequencing process, DNA was shredded, followed by end-repair, dA-tailing, multiplex adapter ligation and purification. The prepared library was sequenced in Illumina Novaseq 6000 (Illumina, San Diego, CA, USA) platform, with 150 bp paired-end reads and 10.72 Gb raw data was generated. The sequencing reads were quality checked by FastQC (0.12.1)^93^, which showed Phred score > 25, and 35.7 million short reads at each directions. Quality trimming was performed using TrimGalore (0.6.6)^94^, which auto-detected Illumina adaptors and removed 5 bp bases from both 3’ and 5’ ends. Quality trimming trimmed out only 0.3% reads and retained high confidence reads for the assembly.

### Mitogenome assembly and annotation

The mitogenome of *O. pama* was assembled using *De-Novo* assembler NOVOPlasty v4.3.1^95^. Assembly type was set as mito, genome range set up between 15,000 and 18,000 and 33 K-mer was used in the config file of NOVOplasty. Further, the assembly was manually checked in CLC Genome Browser^96^ with length and similarity fractions set to 0.8 and 0.9, respectively, along with other parameters in default mode. Mitochondrial genes were annotated using Mitos Web Server and MitoZ (v3.6)^97,98^. While annotation from both programs agreed, we kept them. While disagreed, the annotations were manually validated using multiple sequence alignment of 30 species, belonging to the same family Sciaenidae. Multiple sequence alignment was performed on MEGA 11 software using ClustalW algorithm. The gap opening and extension penalties were 15.00 and 6.66, respectively, along with IUB DNA weight matrix, 0.50 transition weight and, 30% delay divergent cutoff.

The species names and associated accession numbers are shown in Supplementary Table S2.

The alignments of the homologous sequence revealed that some features of the mitogenome (*16S rRNA, nad6* and CR) were incorrectly annotated by annotation tools which was solved by manual annotation. The tRNA genes were annotated with Mitos Web Server and tRNA scan-SE 2.0^99^. All the PCGs were translated to protein frame 1 by ExPaSY translate tool^100^. The circular map of the mitogenome of *O. pama* was drawn using GenomeVX^101^. To explore various features of *O. pama* mitogenome, different online tools were utilized. The ETAS and conserved sequence blocks of *O. pama* were located by performing multiple sequence alignment (MSA) of the CR with the respective regions of 30 other Sciaenid species and comparing the results with the ETAS and CSB regions of *M miiuy*^41^ and *L. crocea*^42^. MSA was conducted using ClustalW^102^ in MEGA 11^103^ software. The base composition of the mitogenome was estimated using online tool VectorBuilder and skewness was calculated in Microsoft Exel using the formula: AT-skew = (A-T)/(A + T); GC-skew = (G-C)/(G + C)^104^. Relative synonymous codon usage (RSCU) of the PCGs were estimated by CAI (Codon Adaptation Index) calculator^105^. The graphics had been constructed using ggplot2 in the tidyverse R package^106^ (See “Data availability” section).

### Comparative genomics and phylogeny

The complete mitochondrial genomes of 31 sciaenids were used to conduct a comparative mitogenomics study of Sciaenidae. On August 16, 2023, a manual search on NCBI was carried out with the search term “Sciaenidae complete mitochondrion” to collect all the complete mitochondrial genomes available for different species of Sciaenidae. The mitogenomes were compared through MSA using MEGA 11^103^. To compare PCGs across all mitogenomes, non-protein coding sequences were cleaved manually and concatenated into one sequence for each mitogenome. The sequences undergone RSCU value measurement using the same methods described above. Due to the presence of ambiguous base pairs, two species, namely – *M. littoralis* and *P. diacanthus*, were excluded from downstream RSCU analysis. Based on the RSCU values, an unsupervised hierarchical agglomerative clustering algorithm, with Euclidian dissimilarity matrix was applied using hclust function of Cluster R package^107^. Before constructing clustering dendrogram, four different linkage methods, i.e. Complete, Single, Average and Ward, were evaluated and Ward method showed the strongest clustering structure (0.96), followed by complete (0.94), average (0.88) and single (0.87). Plot function of the same package was used to construct the RSCU clustering dendrogram.

Each of the 13 PCGs, two rRNAs and the CR region were separately aligned using ClustalW^102^ of MEGA 11^103^. The conserved-variable site ratio, K2P distance, Ka/Ks substitution ratio of 13 PCGs were calculated using MEGA 11^103^.

A maximum likelihood tree was constructed with 1000 bootstrap replications and K2P model for nucleotide substitution using MEGA 11^103^. The complete mitochondrial genome of total 30 species were selected including *S. laticaudus* (Order: Carcharhiniformes) as an outgroup. The sequences were aligned using ClustalW (using default parameters). Non-shared, loose ends of sequences were trimmed manually, to keep shared alignment region within the alignment. Nearest-Neighbor-Interchange (NNI) was used as tree inference and number of threads were 3. The number of Bootstrap-replication was chosen 1000 for node reliability.

### Supplementary Information

Supplementary Information.

## Data Availability

The data generated and analyzed during the current study are available in the NCBI GenBank database. (https://www.ncbi.nlm.nih.gov/nuccore/OQ784575.1). The R codes contain all datasets and codes, (including the Hierarchical clustering), which we used to construct the figures are available in the following repository https://github.com/asifratul/otolithoides_pama.

## Abbreviations

PCG: Protein coding gene
*nad1–6* and nad4l: NADH dehydrogenase subunit 1–6 and 4l
*cox1-3*: Cytochrome c oxidase subunits 1–3, respectively
*atp6* and * atp8*: ATPase subunit 6 and 8, respectively
*cytb*: Cytochrome b
rRNA: Ribosomal RNA
tRNA: Transfer RNA
DHU: Dihydrouracil
CR: Putative control region
ETAS: Extended termination-associated sequence
CSB: Conserved sequence block
O_L_: Origin of Light strand replication
mitogenome: Mitochondrial genome
bp: Base pair
IGS: Intergenic spacer
RSCU: Relative synonymous codon usage
Ka/Ks: Non-synonymous and synonymous substitution ratio
ML: Maximum likelihood
CTAB: Cetyltrimethylammonium bromide
NCBI: National Center for Biotechnology Information
BLAST: Basic Local Alignment Search Tool
