# Supplementary material for: Complete mitogenome and intra-family comparative mitogenomics showed distinct position of Pama Croaker Otolithoides pama
Source: Sci Rep. 2024 Jun 15;14:13820. doi: 10.1038/s41598-024-64791-1 (PMC11180200; doi:10.1038/s41598-024-64791-1)
Supplement: Supplementary file 1 — Supplementary Information. [file 41598_2024_64791_MOESM1_ESM.pdf]

Complete mitogenome and intra-family comparative mitogenomics showed distinct position of Pama Croaker *Otolithoides pama*

Most Ayesha Siddika[1], Khandaker Asif Ahmed[1,2], Mohammad Shamimul Alam[1*], Jannatul Bushra[1] and Rowshan Ara Begum[1]

[1]Genetics and Molecular Biology Laboratory, Department of Zoology, University of Dhaka, Dhaka-1000, Bangladesh.

[2]Australian Animal Health Laboratory (AAHL), CSIRO, East Geelong, Australia.

*Corresponding author: shamimul@du.ac.bd

Supplementary Table S1. Nucleotide composition of mitochondrial genes of *O. pama*.
CR=control region, PCG=protein coding gene.

Gene	Total bases	Number of individual bases				Percentage of bases (%)				Percentage content	
		A	T	G	C	A	T	G	C	AT%	GC%
tRNA^{Phe} (F)	69	27	13	14	15	39.13	18.84	20.29	21.74	57.97	42.03
12S rRNA	950	306	194	196	254	32.21	20.42	20.63	26.74	52.63	47.40
tRNA^{Val} (V)	72	23	14	15	20	31.94	19.44	20.83	27.78	51.38	48.61
16S rRNA	1703	578	360	315	450	33.94	21.14	18.50	26.42	55.08	44.90
tRNA$^{Leu\ (UUR)}$	74	20	18	16	20	27.03	24.32	21.62	27.03	51.35	48.65
nad1	975	262	276	127	310	26.87	28.31	13.03	31.79	55.18	44.82
tRNA^{Ile} (I)	70	22	18	15	15	31.43	25.71	21.43	21.43	57.14	42.86
tRNA^{Gln} (Q)	71	14	23	21	13	19.72	32.39	29.58	18.31	52.11	47.89
tRNA^{Met} (M)	69	21	21	10	17	30.43	30.43	14.49	24.64	60.86	39.13
nad2	1047	298	274	107	368	28.46	26.17	10.22	35.15	54.63	45.40
tRNA^{Trp} (W)	72	23	13	17	19	31.94	18.06	23.61	26.39	50.00	50.00
tRNA^{Ala} (A)	69	17	25	17	10	24.64	36.23	24.64	14.49	60.87	39.13
tRNA^{Asn} (N)	73	13	23	24	13	17.81	31.51	32.88	17.81	49.32	50.69
tRNA^{Cys} (C)	66	18	17	18	13	27.27	25.76	27.27	19.70	53.03	46.97
tRNA^{Tyr} (Y)	70	16	21	20	13	22.86	30.00	28.57	18.57	52.86	47.14
cox1	1557	396	470	272	419	25.43	30.19	17.47	26.91	55.62	44.38
tRNA$^{Ser(UCN)}$	71	18	22	18	23	25.35	30.99	25.35	18.31	56.34	43.66
tRNA^{Asp} (D)	69	21	18	13	17	30.43	26.09	18.84	24.64	56.52	43.48
cox2	691	205	187	103	196	29.67	27.06	14.91	28.36	56.73	43.27
tRNA^{Lys} (K)	74	23	17	16	18	31.08	22.97	21.62	24.32	54.05	45.94
atp8	168	65	48	11	44	38.69	28.57	06.55	26.19	67.26	32.74
atp6	684	193	195	76	220	28.22	28.51	11.11	32.16	56.73	43.27
cox3	786	202	220	121	243	25.70	27.99	15.39	30.92	53.69	46.31
tRNA^{Gly} (G)	71	26	23	11	11	36.62	32.39	15.49	15.49	69.01	30.98
nad3	349	86	110	47	106	24.64	31.52	13.47	30.37	56.16	43.84
tRNA^{Arg} (R)	69	26	21	10	12	37.68	30.43	14.49	17.39	68.11	31.88
nad4l	297	73	76	37	111	24.58	25.59	12.46	37.37	50.17	49.83
nad4	1381	388	382	177	434	28.10	27.66	12.82	31.43	55.76	44.25
tRNA^{His} (H)	69	27	18	10	14	39.13	26.09	14.49	20.29	65.22	34.78
tRNA$^{Ser(AGY)}$	68	16	14	17	21	23.53	20.59	25.00	30.88	44.12	55.88
tRNA$^{Leu(CUN)}$	73	24	18	14	17	32.88	24.66	19.18	23.29	57.54	42.47
nad5	1839	552	493	211	583	30.02	26.81	11.47	31.70	56.83	43.17
nad6	522	70	212	185	55	13.41	40.61	35.44	10.54	54.02	45.98
tRNA^{Glu} (E)	69	17	24	18	10	24.64	34.78	26.09	14.49	59.42	40.58
cytb	1141	310	315	144	372	27.17	27.61	12.62	32.60	54.78	45.22
tRNA^{Thr} (T)	72	13	18	19	22	18.06	25.00	26.39	30.56	43.06	56.95
tRNA^{Pro} (P)	70	17	25	19	09	24.29	35.71	27.14	12.86	60.00	40.00
CR	835	268	268	124	175	32.10	32.10	14.85	20.96	64.20	35.81
PCGs	11437	3242	3116	1488	3591	28.35	27.24	13.01	31.40	55.60	44.40
Mitogenome	16513	4892	4323	2417	4881	29.63	26.18	14.64	29.56	55.81	44.20

Supplementary Table S2. List of Sciaenidae species utilized in the comparative mitogenomics study. A list of 30 species of Sciaeniade family with verified full length mitochondrial genome collected from the NCBI GenBank database. Multiple sequence alignment of each gene of 30 spp. including *O. pama* was done to cross check genome annotation and performing phylogenetic study.

SL. No.	Species	Mitogenome Length (bp)	Accession number	Country
1	*Argyrosomus japonicus*	16,496	NC_017610.1	Zhejiang, China
2	*Argyrosomus amoyensis*	16,490	NC_025937.1	Guangdong, China
3	*Johnius belangerii*	19,154	NC_022464.1	Zhejiang, China
4	*Johnius borneensis*	18,630	NC_041308.1	Fujian, China
5	*Johnius carouna*	18,752	NC_035981.1	Fujian, China
6	*Johnius grypotus*	18,523	NC_021130.1	Shandong, China
7	*Pennahia argentata*	16,485	NC_015202.1	Zhejiang, China
8	*Pennahia pawak*	16,408	NC_035942.1	Fujian, China
9	*Pennahia macrocephalus*	16,508	NC_031409.1	Fujian, China
10	*Collichthys niveatus*	16,469	NC_014263.1	Zhejiang, China
11	*Collichthys lucidus*	16,442	NC_014350.1	Zhejiang, China
12	*Larimichthys crocea*	16,466	NC_011710.1	Shandong, China
13	*Larimichthys polyactis*	16,470	NC_013754.1	Shandong, China
14	*Bahaba taipingensis*	16,500	NC_018347.1	Shandong, China
15	*Atrobucca nibe*	16,842	NC_035982.1	Fujian, China
16	*Otolithes ruber*	16,589	NC_033909.1	Fujian, China
17	*Aplodinotus grunniens*	16,487	MG599474.1	Jiangsu, China
18	*Chrysochir aureus*	16,505	NC_016987.1	Zhejiang, China
19	*Sciaenops ocellatus*	16,500	NC_016867.1	Zhejiang, China
20	*Miichthys miiuy*	16,493	NC_014351.1	Zhejiang, China
21	*Dendrophysa russelii*	16,626	NC_017606.1	Zhejiang, China
22	*Nibea albiflora*	16,499	NC_015205.1	Zhejiang, China
23	*Nibea coibor*	16,502	NC_025307.1	Guangdong, China
24	*Nibea miichthioides*	16,490	NC_029875.1	Zhejiang, China
25	*Nibea mitsukurii*	16,498	NC_034350.1	Chiba, Japan
26	*Protonibea diacanthus*	16,521	NC_024573.1	Darwin, Australia
27	*Menticirrhus littoralis*	16,499	MT199157.1	Para, Brasil
28	*Micropogonias furnieri*	16,496	MW646294.1	Asan, Korea
29	*Pseudotolithus elongatus*	16,497	NC_044717.1	Busan, Korea
30	*Pseudotolithus typus*	16,502	NC_056258.1	Busan, Korea

Supplementary Figure S1. Secondary structures of tRNAs of *O. pama*. a) Putative secondary structure of 22 tRNAs and b) secondary structure of tRNA$^{Ser1\ (AGY)}$.

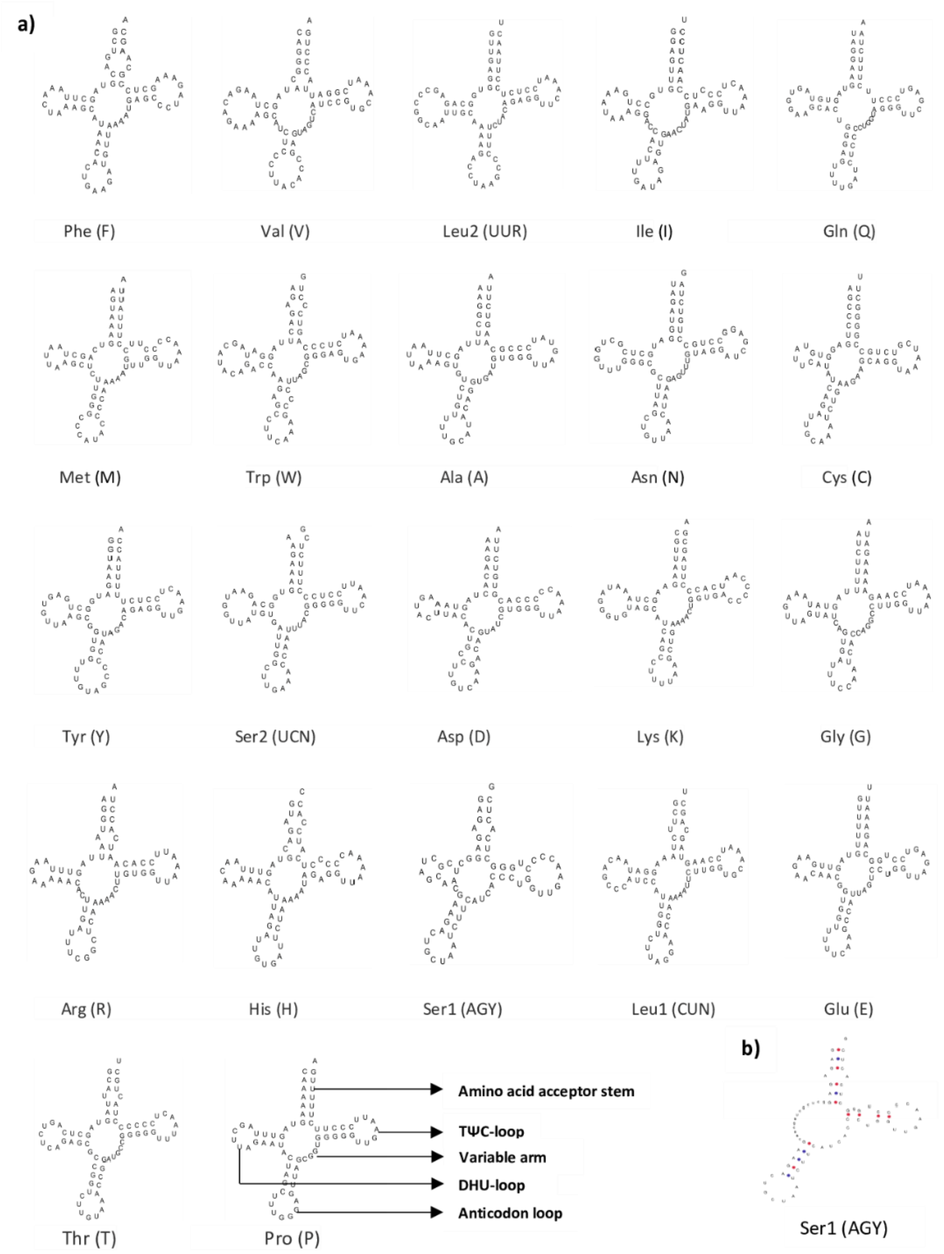

Supplementary Figure S2. A scatter plot showing relationship between the percentages of AT (left) and GC content (right) and their corresponding skewness within different mitochondrial gene groups of *O. pama*

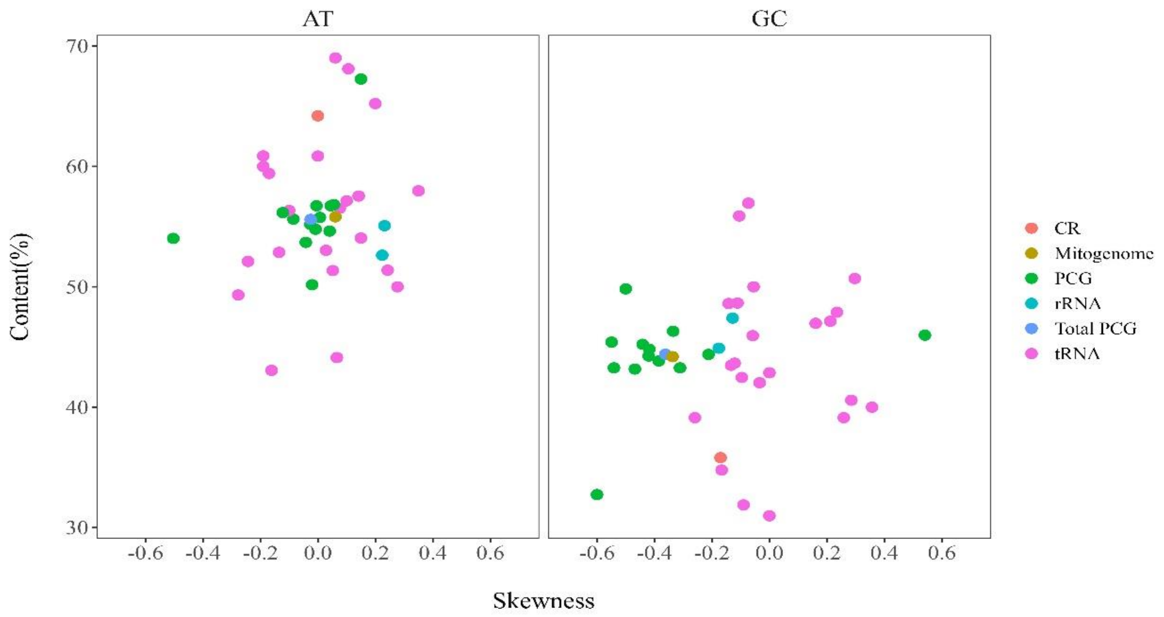

Supplementary Figure S3. The phylogenetic tree of the family Sciaenidae, based on Protein coding genes (PCG) only.

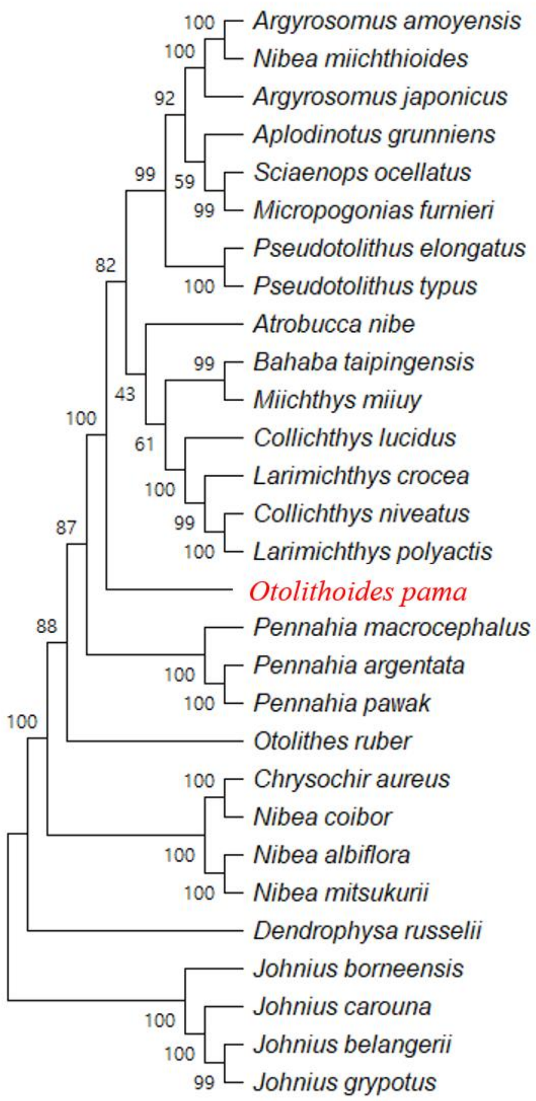

Supplementary Figure S4. Hierarchical agglomerative clustering analysis, based on RSCU values across PCG codons of Sciaenid mitogenomes. Euclidian dissimilarity matrix, and average linkage method were used to construct the dendogram. *Menticirrhus littoralis* and *Protonibea diacanthus* mitogenomes were excluded, due to presence of ambiguous bases.

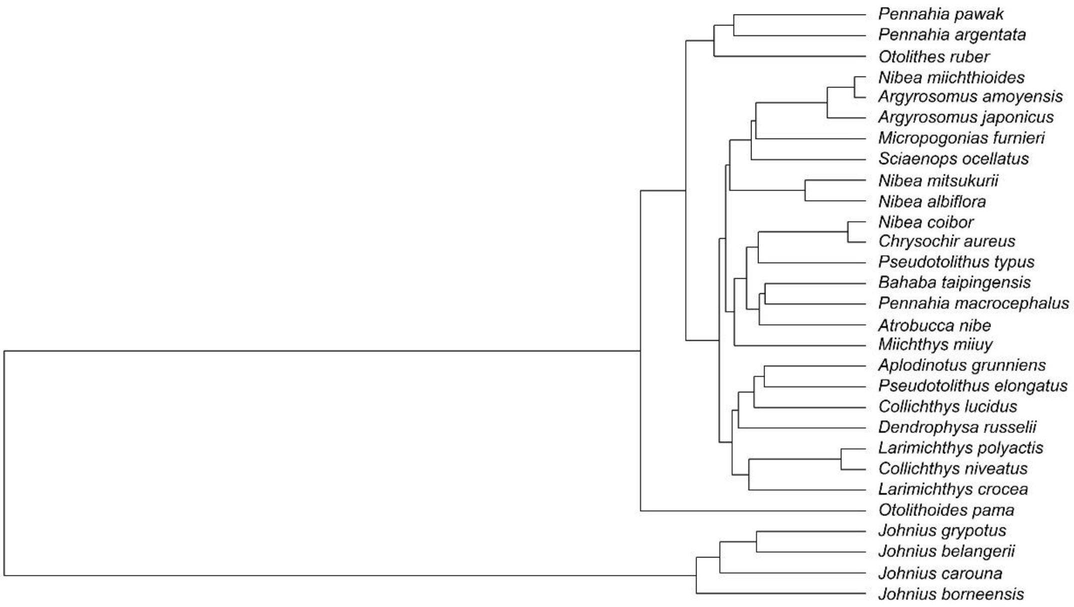

Supplementary Data 1

>Otolithoides_pama *cox1* partial sequence

GGCACAGCCTTAAGCCTCCTAATCCGGGCGGAACTCAGCCAACCCGGCTCACTCCTAGGAGATGACCAAATTT
TTAATGTAATTGTTACAGCACATGCCTTCGTCATAATCTTCTTTATAGTAATACCTGTAATAATTGGAGGATTTG
GGAACTGGCTTGTACCTCTAATAATTGGAGCCCCTGACATAGCATTTCCCCGAATAAACAACATAAGCTTCTGA
CTCCTTCCTCCTTCTTTTTTACTACTCCTGACCTCTTCAGGGGTAGAGGCAGGTGCTGGAACAGGATGAACCGT
TTATCCCCCTCTTGCTGGAAATCTCGCACACGCAGGAGCTTCTGTGGACCTAGCCATTTTTTCCCTCCACCTGGC
AGGTGTTTCATCAATTCTTGGGGCCATCAACTTTATTACAACTATTATTAATATAAAACCCCCAGCCATCTCCCA
ATACCAAACACCTCTGTTCGTATGGGCCGTTCTTATTACAGCAGTCCTCCTACTTCTCTCGCTACCAGTATTAGC
TGCTGGTATTACAATACTTTTAACAGACCGTAACCTAAATACAACTTTCTTCGACCCTGCAGGAGGGGGAGAC
CCAAT
